# Draft Genome Sequence of the d-Xylose-Fermenting Yeast *Spathaspora xylofermentans* UFMG-HMD23.3

**DOI:** 10.1128/genomeA.00815-17

**Published:** 2017-08-17

**Authors:** Daiane D. Lopes, Samuel P. Cibulski, Fabiana Q. Mayer, Franciele M. Siqueira, Carlos A. Rosa, Ronald E. Hector, Marco Antônio Z. Ayub

**Affiliations:** a Biotechnology and Biochemical Engineering Laboratory (BiotecLab), Science and Food Technology Institute, Federal University of Rio Grande do Sul, Porto Alegre, Rio Grande do Sul, Brazil; b Veterinary Research Institute, Fepagro Animal Health, Eldorado do Sul, Rio Grande do Sul, Brazil; c Centro de Biotecnologia, Federal University of Rio Grande do Sul, Porto Alegre, Rio Grande do Sul, Brazil; d Department of Microbiology, Biological Science Institute, Federal University of Minas Gerais, Belo Horizonte, Minas Gerais, Brazil; e Bioenergy Research Unit, National Center for Agricultural Utilization Research, USDA-ARS, Peoria, Illinois, USA

## Abstract

Here, we report the draft genome sequence of the yeast *Spathaspora xylofermentans* UFMG-HMD23.3 (=CBS 12681), a d-xylose-fermenting yeast isolated from the Amazonian forest. The genome consists of 298 contigs, with a total size of 15.1 Mb, including the mitochondrial genome, and 5,948 predicted genes.

## GENOME ANNOUNCEMENT

*Spathaspora xylofermentans* UFMG-HMD23.3 (=CBS 12681) is an asexual d-xylose-fermenting yeast isolated from rotting wood of the Amazonian environment in Brazil (1). Species of the *Spathaspora* clade are known for their ability to convert xylose to ethanol and have the potential for lignocellulosic ethanol production (2–6). Limitations in lignocellulosic ethanol production associated with poor xylose assimilation by engineered *Saccharomyces cerevisiae* strains could be solved through knowledge about the mechanisms for xylose fermentation in natural, or wild-type, yeasts (7–10). Considering the importance of the genus *Spathaspora* and the species closely related to this clade, we proceeded to annotate the genomic information of the strain presented here. The genomic information from this yeast will contribute to advancing technologies to efficiently produce lignocellulosic-based ethanol, the so-called second-generation ethanol, either by the direct use of a genetically improved strain or as a source of genes needed for xylose fermentation in genetically modified industrial strains of *S. cerevisiae*.

*S. xylofermentans* DNA was isolated using the Wizard genomic DNA purification kit (Promega). DNA libraries were prepared with a Nextera DNA library prep kit (Illumina) and sequenced in the MiSeq system (Illumina) (paired-end, 500-cycle version 2 kit). The raw sequence data comprise 3,827,910 high-quality paired-end reads. Reads were imported into CLC Genomics Workbench version 10, trimmed, and *de novo* assembled. Gene prediction was performed with AUGUSTUS (11), and genome statistics were generated by QUAST (12). The genome of *S. xylofermentans* HMD23.3 consists of 293 contigs (largest contig, 639,790 bp; *N*_50_, 142,604 bp), with a total size of 15,098,813 bp (mean coverage, ∼55×) and a G+C content of 35.34%. Among 5,948 potential protein-coding genes, 92.8% encode proteins with assigned functional roles and showed similarity to yeast species of the CTG clade, mainly to *S. passalidarum* strain NRRL Y-27907 (13). The mitochondrial DNA was assembled into a 23,201-bp fragment (contig 138, mean coverage of 483×). tRNAscan-SE (14) predicted 249 tRNA genes scattered across the contigs. RNAmmer (15) identified 28S, 18S, and 5.8S rRNA genes at contig 178.

*S. xylofermentans* HMD23.3 has genes required for xylose assimilation and fermentation, which are important for lignocellulosic-based ethanol production. Genes for conversion of d-xylose to d-xylulose (*XYL1* and *XYL2*) and xylulokinase for incorporation of d-xylulose-5P into the pentose phosphate pathway were identified at contigs 27, 110, and 124. Only one *XYL1* gene was identified (contig 124), and the Xyl1p is 93% and 76% identical to Xyl1.1p and Xyl1.2p of *S. passalidarum* NRRL Y-27907, respectively. Xyl1.2p showed a preference for NADH over NADPH in activity tests of xylose reductase, which allows for the anaerobic fermentation of xylose (7). At least 21 sugar transporters were identified, and some of them were related as possible xylose transporters.

### Accession number(s).

This whole-genome shotgun project has been deposited at DDBJ/EMBL/GenBank under the accession no. NDXA00000000. The version described in this paper is the first version, NDXA01000000.
